# A novel approach for preparation of the antisera reagent for potency determination of inactivated H7N9 influenza vaccines

**DOI:** 10.1111/irv.12365

**Published:** 2016-01-29

**Authors:** Falko Schmeisser, Xianghong Jing, Manju Joshi, Anupama Vasudevan, Jackeline Soto, Xing Li, Anil Choudhary, Noel Baichoo, Josephine Resnick, Zhiping Ye, William McCormick, Jerry P. Weir

**Affiliations:** ^1^ Laboratory of DNA VirusesDivision of Viral ProductsCenter for Biologics Evaluations and Research, Food and Drug AdministrationSilver SpringMDUSA; ^2^ Laboratory of Respiratory Viral DiseasesDivision of Viral ProductsCenter for Biologics Evaluations and Research, Food and Drug AdministrationSilver SpringMDUSA; ^3^ Division of Biological Standards and Quality ControlCenter for Biologics Evaluations and Research, Food and Drug AdministrationSilver SpringMDUSA

**Keywords:** Influenza vaccine reagents, influenza vaccines, single‐radial immunodiffusion assay, vaccine potency assay

## Abstract

**Background:**

The potency of inactivated influenza vaccines is determined using a single‐radial immunodiffusion (SRID) assay and requires standardized reagents consisting of a Reference Antigen and an influenza strain‐specific antiserum. Timely availability of reagents is a critical step in influenza vaccine production, and the need for backup approaches for reagent preparation is an important component of pandemic preparedness.

**Objectives:**

When novel H7N9 viruses emerged in China in 2013, candidate inactivated H7N9 influenza vaccines were developed for evaluation in clinical trials, and reagents were needed to measure vaccine potency.

**Methods:**

We previously described an alternative approach for generating strain‐specific potency antisera, utilizing modified vaccinia virus Ankara vectors to produce influenza hemagglutinin (HA)‐containing virus‐like particles (VLPs) for immunization. Vector‐produced HA antigen is not dependent upon the success of the traditional bromelain‐digestion and HA purification.

**Results:**

Antiserum for H7N9 vaccines, produced after immunization of sheep with preparations of bromelain‐HA (br‐HA), was not optimal for the SRID assay, and the supply of antiserum was limited. However, antiserum obtained from sheep boosted with VLPs containing H7 HA greatly improved the ring quality in the SRID assay. Importantly, this antiserum worked well with both egg‐ and cell‐derived antigen and was distributed to vaccine manufacturers.

**Conclusions:**

Utilizing a previously developed approach for preparing vaccine potency antiserum, we have addressed a major bottleneck encountered in preparation of H7N9 vaccine reagents. The combination of br‐HA and mammalian VLPs for sequential immunization represents the first use of an alternative approach for producing an influenza vaccine potency antiserum.

## Introduction

The traditional assay used to measure potency of inactivated influenza vaccines is a single‐radial immunodiffusion (SRID) assay that utilizes an influenza strain‐specific antibody to measure the content of virus hemagglutinin (HA) in the vaccine by comparison to a homologous HA Reference Antigen.^1^, ^2^ The antigen and antibody components of the assay constitute the potency reagents that are prepared and distributed by regulatory agencies for use by regulators and vaccine manufacturers to determine the potency of licensed inactivated influenza vaccines, and thereby ensure standardization of vaccines made by various manufacturers.

Previously, we described an alternative approach for generating strain‐specific potency antibody for use in potency assays for inactivated influenza virus vaccines.^3^ The strategy involved construction of DNA and modified vaccinia virus Ankara (MVA) vectors that expressed HA. The HA‐expressing vectors could be used directly for immunization of animals, but the MVA vector system was also an efficient method for producing influenza HA virus‐like particles (VLPs) that could be used as an antigen for immunization. Whereas the traditional approach to producing potency antibody involves virus purification, bromelain treatment to remove the HA from the virus particle,^4^, ^5^ and finally purification of the br‐HA for animal immunization, HA‐expressing vectors can be constructed in the absence of influenza virus, and the vector‐produced HA antigen for immunization is not dependent upon the success of the bromelain‐digestion and HA purification procedure. Demonstration of the feasibility of this novel method for potency antiserum production was important, as it presented a viable alternative method for one of the potential bottlenecks in inactivated pandemic influenza vaccine production, namely the production of potency reagents.

In 2013, novel H7N9 viruses emerged in China causing severe respiratory disease in humans with several hundred fatalities by early 2015 (http://www.who.int/influenza/human_animal_interface/HAI_Risk_Assessment/en/).^6^, ^7^ Because of the serious nature of the outbreak, as well as the paucity of data regarding immunogenicity and the nature of a protective immune response to H7N9 viruses, candidate H7N9 inactivated vaccines were developed by manufacturers for evaluation in clinical trials.^8^, ^9^ As for all inactivated influenza virus vaccines, strain‐specific reagents were needed to measure vaccine potency, and work commenced in regulatory laboratories to prepare and calibrate the necessary reagents.

Here, we describe some of the difficulties encountered in preparation of the potency antiserum for H7N9 inactivated vaccines, and how the previously developed alternative method for generating strain‐specific potency antibody was successfully used to generate a potency antiserum for these vaccines. Virus‐like particles, generated in mammalian cells using MVA vectors expressing the A/Shanghai/2/2013 HA, substantially boosted the H7 HA‐specific antibody response in sheep. The resulting antisera were suitable for use in vaccine potency SRID assays with Reference Antigen standards prepared in either embryonated eggs or mammalian cell culture and were used to quantify the HA content in H7N9 vaccines. This report documents the novel process used to prepare the H7N9 vaccine potency antisera, and the results reinforce the importance of having alternative strategies available to address potential bottlenecks in influenza vaccine manufacturing as part of pandemic preparedness.

## Materials and methods

### Cells and viruses

Influenza viruses were propagated in 9‐day‐old specific pathogen‐free embryonated chicken eggs. All H7 influenza viruses used in the study are candidate vaccine viruses (http://www.who.int/influenza/vaccines/virus/en/). H7N3 vaccine virus A/mallard/Netherlands/12/2000 (NIBRG‐60) was developed by NIBSC (UK); H7N9 vaccine virus A/Shanghai/2/2013 (IDCDC‐RG32A) was developed by CDC (USA); H7N9 vaccine virus A/Shanghai/2/2013 (CBER‐RG4A) was developed at CBER/FDA (USA).

### Preparation of bromelain‐cleaved HA and VLPs for immunization

Bromelain‐cleaved HA was prepared essentially as described previously.^10^ Briefly, viruses were grown in 100 embryonated chicken eggs at 33°C by inoculation of 0·2 ml of diluted virus stock containing ~10^4^ pfu and purified. Purified virus was diluted to a concentration of 10 mg/ml and incubated with bromelain, followed by br‐HA purification through a continuous sucrose gradient. Virus‐like particles containing influenza A/Shanghai/2/2013 HA were prepared as described.^11^

### Animals and immunizations

All immunizations and blood draws were performed in accordance with an animal protocol approved by the Institutional Animal Care and Use Committees of the Center of Biologics Evaluation and Research (CBER) and the National Institutes of Health. Four sheep were used in this study. The primary immunization used ~90 μg br‐HA mixed with complete Freund's adjuvant (CFA). Two weeks later, sheep were boosted with a ~50 μg of br‐HA in incomplete Freund's adjuvant (IFA). An additional boost of all sheep with br‐HA was performed ~3 weeks later. In addition, SH830 and SH851 were boosted with influenza H7 (A/Shanghai/02/2013) VLPs in IFA. Plasma was collected and sera were generated following defibrination; successive bleeds from individual sheep were evaluated and pooled to facilitate preparation of larger lots of reference antiserum. The sheep were maintained for extended periods of time and were periodically boosted with VLP to maintain the anti‐HA titers. Two sheep (SH839 and SH845), which responded poorly to the initial immunizations, were not boosted with VLPs.

### SRID assay

The SRID assay was performed essentially as described previously^2^, ^12^ and is based on the diffusion of detergent‐disrupted antigen (vaccine or virus reference standard) into an agarose gel containing specific HA antibodies. Briefly, 1% agarose (Lonza, Walkersville, MD, USA) was prepared in PBS and the optimal amount of HA antibodies added. Gels were cast on GelBond Film (Lonza), and four millimeter wells were punched into the solidified gel. For quantitative data analysis, antigens were diluted, treated with Zwittergent 3‐14 (Calbiochem; EMD Biosciences, La Jolla, CA, USA) to a final concentration of 1%, and incubated for 30 minutes at room temperature. Dilutions of detergent‐treated samples were loaded sequentially onto the agarose gel and gels were incubated at room temperature for 18–24 hours in a humidified chamber to allow diffusion of the antigens. Following incubation, gels were washed sequentially with a saline solution and purified water, dried, and stained with Coomassie Brilliant Blue. All dried gels were scanned for image evaluation. For quantitative analysis, two perpendicular diameters of the precipitin rings were measured using an Immulab system (GT Vision, Hagerstown, MD, USA), and the concentration of HA in the sample was calculated by generating a dose–response curve against a Reference Antigen of pre‐determined HA concentration from the linear region of the parallel dose–response curve. Standard agarose gels were 3 mm thick; however, in some experiments, thin gels (i.e., 2·2 mm) were prepared to facilitate better visualization of precipitin rings.

## Results

### Preparation and purification of bromelain‐HA from H7N3 A/mallard/Netherlands/12/2000 and H7N9 A/Shanghai/2/2013 for sheep immunization

Even before the emergence of the H7N9 influenza virus in China in 2013, several candidate H7 inactivated vaccines had been prepared and evaluated in clinical trials.^13^, ^14^, ^15^ Reagents to assess vaccine potency by the SRID assay, which include inactivated virus as the Reference Antigen and a strain‐specific antiserum, were successfully prepared by traditional methods for evaluation of those candidate vaccines. This suggested that a similar approach to preparation of vaccine potency reagents might be successful for H7N9 vaccines. Consequently, as work progressed to develop pilot vaccine lots, we began preparation of an antiserum reagent for use in the SRID potency assay. In addition to a new H7N9 candidate vaccine virus RG32A containing the HA from A/Shanghai/2/2013 (A/Shanghai), we had available a previous H7N3 vaccine virus NIBRG‐60 containing the HA from A/mallard/Netherlands/12/2000 (A/mallard/NL), an H7 virus of Eurasian origin that is phylogenetically related to the HA from the recent H7N9 viruses from China.

**Figure 1:**
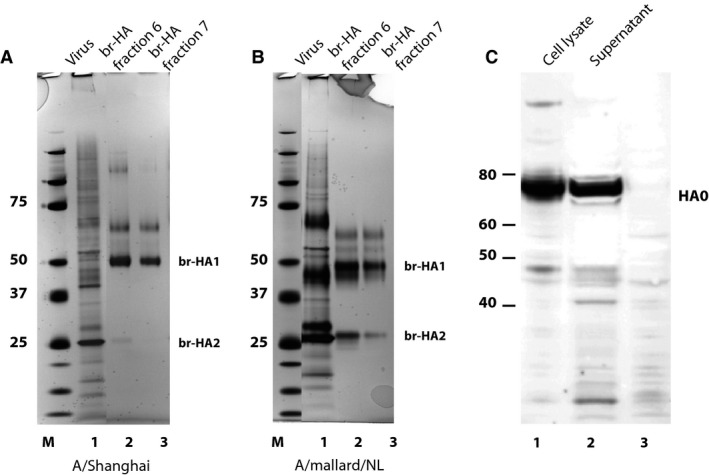
SDS–PAGE analysis of purified br‐HA from H7N9 A/Shanghai/2/2013 and H7N3 A/mallard/Netherlands/12/2000 vaccine viruses. Virus and fractions from the sucrose gradient following bromelain treatment were denatured and subjected to SDS–PAGE under reducing conditions. (A) Virus and br‐HA fractions prepared from H7N9 A/Shanghai/2/2013 vaccine virus. (B) Virus and br‐HA fractions prepared from A/mallard/Netherlands/12/2000 vaccine virus. Lane 1 – purified virus before bromelain treatment; lane 2 – fraction 6 from the 5–20% continuous sucrose gradient; lane 3 – fraction 7. (C) Infected cell extract (lane 1) or supernatant (lane 2) from cells infected with modified vaccinia virus Ankara (MVA) vector expressing H7N9 A/Shanghai/2/2013 HA subjected to SDS–PAGE followed by Western blotting with an H7 monoclonal antibody. Molecular weight markers (M) are shown in kDa.

A/Shanghai and A/mallard/NL candidate vaccine viruses were grown, purified, and treated with bromelain to generate soluble HA trimer that could be used to immunize sheep to prepare antisera. Figure 1 shows an SDS–PAGE analysis of br‐HA preparations from A/Shanghai (Figure 1A) and A/mallard/NL (Figure 1B). In each br‐HA preparation, the predominant species on the gel migrated at the ~50 kDa size expected for HA1, as br‐HA1 should be similar in size to HA1 without bromelain treatment. Higher molecular weight species were observed in both antigen preparations, as well as a smaller band that was the size expected for br‐HA2. At the time when H7 br‐HA was being prepared for sheep immunization, the ability to characterize the quality and purity of the immunogen was limited. An available sheep potency antiserum, prepared several years previously for an H7 A/mallard/NL vaccine, recognized all of the protein bands described above in the SDS–PAGE analysis when used in a Western blot analysis of the br‐HA preparations, but little was known about the specificity or purity of that antiserum (data not shown).

### Antiserum production using H7 bromelain‐HA

**Figure 2:**
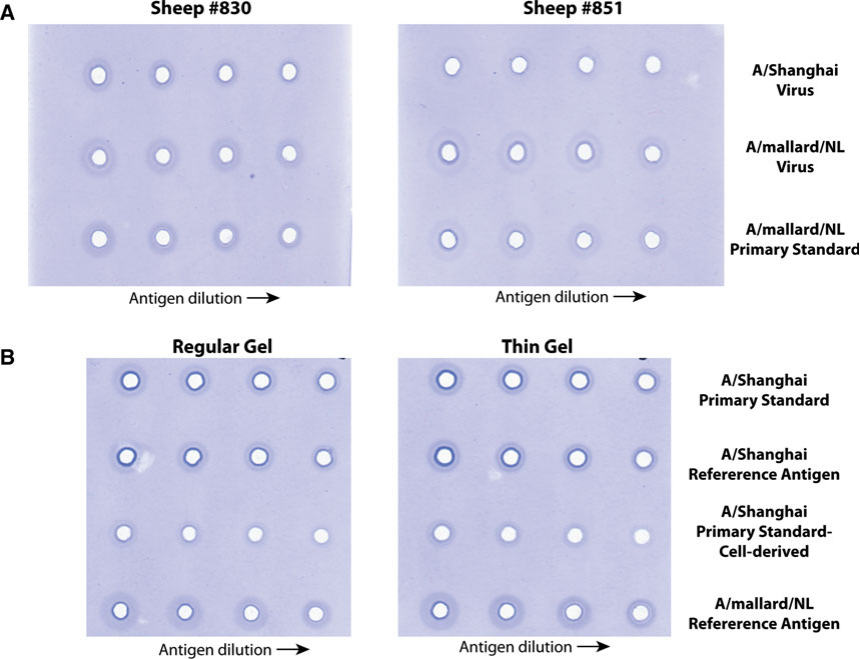
Single‐radial immunodiffusion (SRID) evaluation of sheep polyclonal antiserum produced using br‐HA. (A) Sheep antisera obtained following primary and booster immunization with A/Shanghai/2/2013 br‐HA (#830), or antisera obtained after a primary immunization with br‐HA from A/mallard/Netherlands/12/2000 followed by A/Shanghai/2/2013 br‐HA booster immunization (#851), were analyzed by standard SRID against inactivated A/Shanghai/2/2013 virus (row 1), inactivated A/mallard/Netherlands/12/2000 virus (row 2) or an A/mallard/Netherlands/12/2000 primary standard (row 3). (B) Antiserum from sheep #830 was analyzed by standard and modified (Thin Gel) SRID against primary A/Shanghai/2/2013 antigen standards prepared in eggs (row 1) or cell culture (row 3), and A/Shanghai/2/2013 and A/mallard/Netherlands/12/2000 Reference Antigens (rows 2 and 4, respectively).

Two sheep (SH830 and SH839) were immunized with ~90 μg A/Shanghai br‐HA, and two sheep (SH845 and SH851) were immunized with ~90 μg A/mallard/NL br‐HA using CFA. Three weeks later, all sheep were boosted with 50 μg A/Shanghai br‐HA in IFA. Serum samples were obtained 2 and 3 weeks later and tested for suitability in the SRID assay (Figure 2A). SH830, which received the homologous immunization regimen of A/Shanghai br‐HA, and SH851, which received the heterologous immunization with A/mallard/NL and A/Shanghai br‐HA, were the best responding sheep. Sera from both of these animals showed reactivity in SRID with A/mallard/NL virus and reference standard (rows 2 and 3), but only sera from SH830 sheep appeared to work with A/Shanghai virus (row 1). However, the rings in the SRID assay were faint and not optimal for the assay setup. With a subsequent boost of A/Shanghai br‐HA, the rings improved modestly, but modifications of the SRID assay were still necessary to set up an assay that could be used to calibrate an A/Shanghai Reference Antigen and measure vaccine potency. Figure 2B shows the improvement in ring quality in an SRID assay using thin gels and a relatively high concentration of potency antiserum from Sheep #830. This antiserum was designated as Lot H7‐Ab‐1317 and was subsequently used to calibrate the first A/Shanghai Reference Antigen (#78). However, performance in the SRID assay was still not optimal, and the supply of this antiserum was limited. Also, this potency antiserum did not work well in an SRID with a virus antigen produced in cell culture (Figure 2B, row 3).

### Preparation of mammalian‐derived VLPs containing the influenza A/Shanghai/2/2013 HA

The ability to express high levels of recombinant antigen makes MVA vectors attractive for immunization. In addition, we previously showed that these vectors can be used to produce VLPs containing authentic influenza HA that are good immunogens for production of HA potency antisera,^3^ as well as mouse monoclonal antibodies to HA.^16^ One advantage to this approach for immunization is that the VLPs contain no other influenza proteins other than HA. While work proceeded with the traditional preparation of potency antiserum using br‐HA immunization, we constructed an MVA vector expressing A/Shanghai/2/2013 HA. Western blot analysis verified the expression of A/Shanghai HA of the expected size for HA0 in MVA vector‐infected cells and, as previously observed, a substantial amount of HA was present in the supernatant of vector‐infected cells when the infection was performed with exogenously added neuraminidase (Figure 1C). Following our previously developed protocol,^11^ we prepared A/Shanghai VLPs from the supernatant of Vero cells infected with MVA vector expressing A/Shanghai HA.

### Antiserum production to A/Shanghai H7 HA following VLP boosting

**Figure 3:**
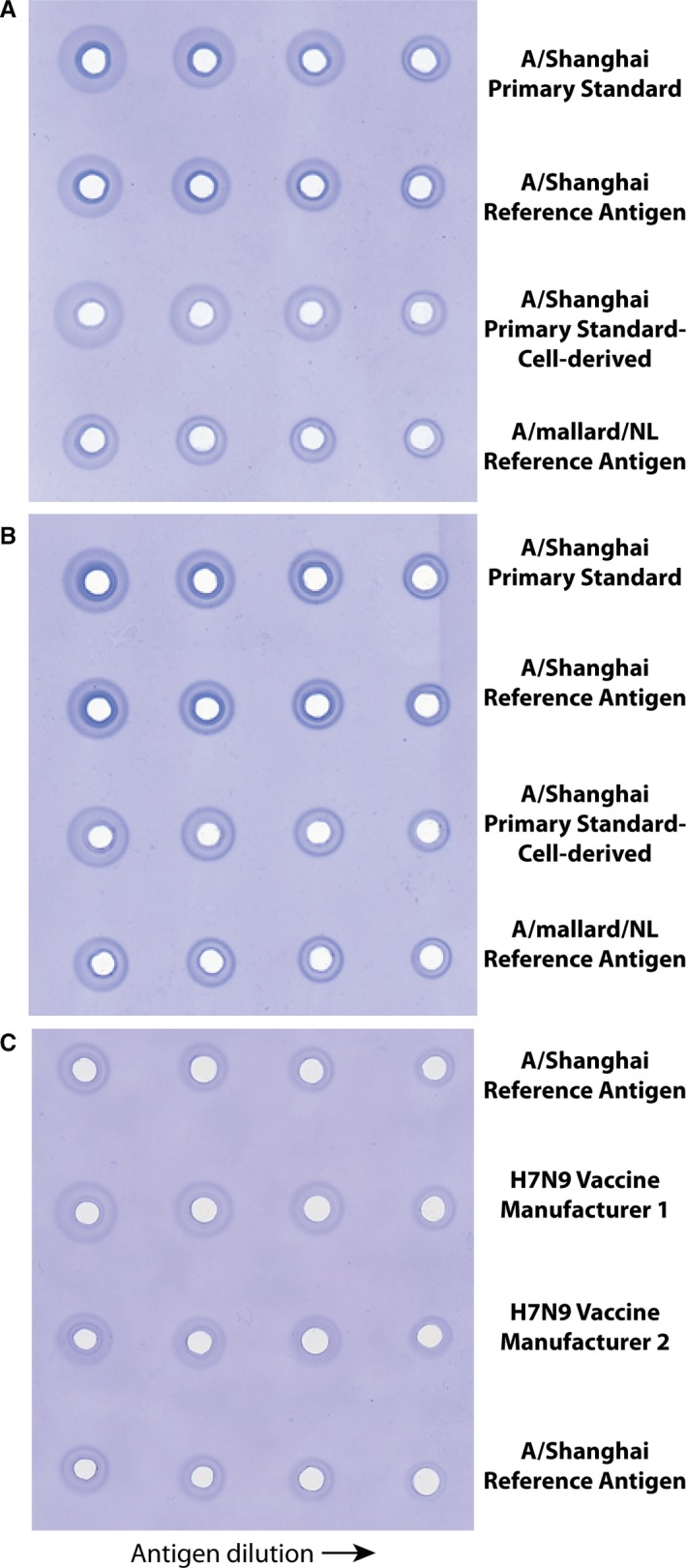
Single‐radial immunodiffusion (SRID) evaluation of sheep polyclonal antiserum following virus‐like particle (VLP) booster immunization. (A) Antiserum from sheep #851 following a booster immunization with H7 A/Shanghai VLPs was analyzed by standard SRID against primary A/Shanghai/2/2013 antigen standards prepared in eggs (row 1) or cell culture (row 3), and A/Shanghai/2/2013 and A/mallard/Netherlands/12/2000 Reference Antigens (rows 2 and 4, respectively). (B) Antiserum from sheep #830 following a booster immunization with H7 A/Shanghai VLPs was analyzed by standard SRID against primary A/Shanghai/2/2013 antigen standards prepared in eggs (row 1) or cell culture (row 3), and A/Shanghai/2/2013 and A/mallard/Netherlands/12/2000 Reference Antigens (rows 2 and 4, respectively). (C) SRID analysis of H7N9 vaccines from two manufacturers using A/Shanghai/2/2013 potency antiserum prepared by an immunization scheme incorporating a booster immunization with H7 A/Shanghai VLPs.

As sera from SH851 had not been especially promising in the initial SRID analyses using A/Shanghai antigen (Figure 2A), we decided to boost this sheep with A/Shanghai VLPs. This immunization (the 4th for SH851) took place approximately 9 weeks after the 3rd immunization. Serum taken 2 weeks following the booster immunization with A/Shanghai VLPs showed a marked improvement in the SRID assay (Figure 3A). The ring quality in the SRID was much improved and the serum worked with both egg‐ (rows 1 and 2) and cell‐derived (row 3) antigen, and modifications to the usual SRID method were not needed. Several bleeds were obtained from Sheep 851 and pooled to produce a substantial quantity (>2000 vials worth) of high quality potency antiserum (Lot designated as H7‐Ab‐1320). In addition, as serum from SH830 was limited, we boosted this sheep with the A/Shanghai VLPs. As for SH851, substantial improvement in the ring quality was obtained, and this antiserum also worked with cell‐derived A/Shanghai antigen (Figure 3B). Multiple bleeds from these two sheep were pooled and several different lots of antiserum were prepared for distribution (totaling >6700 vials). Most importantly, the resulting potency antiserum obtained after VLP boosting was suitable for use in the SRID assay to measure HA content in vaccines. Figure 3C shows a representative SRID gel used to quantify the HA content in influenza H7 vaccines from two different manufacturers relative to the H7 Reference Antigen standard. Taken together, the results demonstrated the successful production of a substantial quantity of potency antisera for measuring the HA content in H7N9 vaccines.

## Discussion

The vast majority of influenza vaccines against either seasonal or pandemic strains of influenza virus are inactivated virus vaccines treated with detergent to enrich the virus HA. Standardization of the potency of these inactivated influenza vaccines, which are produced by many manufacturers throughout the world, is accomplished by use of a common assay, the SRID, and calibrated reagent standards supplied by regulatory agencies. Timely reagent production is always challenging as reagents are strain‐specific and must be produced for each new virus strain in a vaccine, and thus the process is always a potential bottleneck in influenza vaccine production.

The Reference Antigen used in the SRID assay is typically a preparation of whole virus, matching the recommended strain incorporated in the vaccine; the HA content of the Reference Antigen is determined by the World Health Organization Essential Regulatory Laboratories (ERL) in a collaborative exercise. The corresponding strain‐specific antiserum used with the Reference Antigen in the SRID assay is needed in large quantities and is usually prepared by immunization of sheep using soluble HA that has been removed from the virus particle by bromelain treatment. Antiserum produced by immunization with this bromelain‐HA is evaluated for specificity to HA in the SRID assay and its suitability for use in the assay is determined empirically by testing a range of antiserum concentrations to determine the recommended working concentrations that will allow accurate determination of vaccine potency.

Although the procedure for preparing potency antiserum described above is usually reliable, there have been some instances when unexpected difficulties were encountered. For example, the HA of the pandemic H1N1 (2009) virus had a particular sensitivity to bromelain digestion that made purification of virus HA extremely difficult.^3^ Because of the critical nature of reagent preparation and availability in vaccine manufacturing, we previously investigated alternative methods that might be suitable for generating the strain‐specific antiserum reagent. Those studies demonstrated that immunization schemes developed using vectors expressing HA and HA‐containing VLPs produced from such vectors could be used to generate antiserum that was highly strain‐specific and suitable for use in determining the potencies of H5N1 and pandemic H1N1 (2009) vaccines in the SRID assay. HA‐expressing vectors can be generated in the absence of influenza virus availability and VLPs containing HA can be produced without reliance on the success of a bromelain treatment and soluble HA purification procedure. Development and feasibility demonstration of alternative methods for potency antiserum production are important as such methods provide contingency options if difficulties are encountered in traditional methods of reagent preparation.

Immunization of sheep with br‐HA derived from the H7N9 A/Shanghai/2/2013 vaccine virus by the usual protocol immunization protocol yielded sera that was less than ideal for use in the SRID assay. Although modifications could be made to the SRID assay itself to improve the performance of such sera, large concentrations of sera were needed (Figure 2), and the size and intensity of the diffusion rings in the SRID assay were relatively small and faint compared to typical influenza vaccine potency assays. It is not known at this point whether the disappointing results of the traditional br‐HA immunization reflected poor immunogenicity of the H7 HA or resulted from poor purity and/or quantity of the br‐HA in the immunogen preparation. Interestingly, results from some clinical trials of H7 candidate vaccines have suggested that H7 vaccines might be poorly immunogenic,^17^, ^18^ and, in fact, several recent clinical trials have evaluated H7N9 vaccines with adjuvants in an effort to enhance immunogenicity.^19^, ^20^ Regardless, booster immunizations using mammalian VLPs containing HA as the only influenza antigen resulted in marked improvement of H7‐specific antiserum for use in the SRID assay, and these antisera could be used without SRID modification and at more typical antisera concentrations. In addition, the potency antisera obtained using the mammalian VLP boost retained specificity for the H7 subtype in SRID assays (data not shown). Antisera from each sheep were comparatively evaluated throughout the immunization process, using the calibrated Reference Antigen Lot #78 from CBER, as well as cell‐derived antigen. Three antiserum lots were subsequently made using VLP‐boosted sheep sera: H7‐Ab‐1320, H7‐Ab‐1322 and H7‐Ab‐1322a. Together, these lots constituted several thousand vials of potency antisera.

In contrast to previously reported infections with H7 influenza viruses,^21^ the H7N9 viruses that emerged in China in 2013 were extremely severe. The high mortality and the poor understanding of protective immunogenicity of H7 vaccines made development and evaluation of candidate H7N9 vaccines a high public health priority,^8^, ^9^ and reagents to assess vaccine potency were needed to support these clinical trials. The potency antisera described here, prepared using a VLP boost, were suitable for vaccine potency determination of vaccines prepared from two different vaccine manufacturers, demonstrating its general applicability. While only egg‐based vaccines were available to us for potency testing in this study, our results showed that VLP‐boosted potency antiserum also worked well with cell‐derived A/Shanghai antigen, suggesting that it would also be useful for assay of cell‐based vaccines.

Previous studies have demonstrated the feasibility of alternative methods for preparing potency antiserum for the SRID assay, and a recent report describes the development of national reference standards for H7N9 vaccines, including a potency antiserum made by immunization with recombinant HA.^22^ However, the antiserum lots made using the VLP booster immunization that is described in this report were the first influenza vaccine potency antisera distributed by a WHO ERL to vaccine manufacturers for quantifying the HA in their H7N9 vaccines. Thus, this report serves to document the novel approach used to produce this particular vaccine reagent, as well as to provide additional support for the feasibility of using alternative methods for reagent production. Nevertheless, there are a few caveats that should be considered. First, this report describes only a single example of a way to obtain sheep antiserum that otherwise was difficult to generate. Comparative studies, such as using another boost with br‐HA or other preparations of br‐HA, were not possible. At the time the VLP boost was attempted, we did not have additional br‐HA available. Second, the reasons for the failure of the initial sheep immunization have not been thoroughly explored. The immune response from individual sheep is often variable, and the purity and conformational integrity of the br‐HA preparations are difficult to assess, particularly with respect to the time constraints inherent in the reagent preparation process. At the time of H7 potency antiserum preparation, only a very limited number of reagents were available to evaluate the H7 br‐HA (e.g., older H7 potency antiserum). Taken together, it remains difficult to predict when and under what circumstances the traditional br‐HA approach might not be successful in reagent preparation. Nevertheless, the success of the approach taken here for the H7N9 antiserum reagent, even in light of the afore mentioned caveats, emphasizes the importance of having alternative strategies available to address potential bottlenecks in vaccine reagent preparation.

In summary, we have utilized a previously described mammalian VLP production approach for preparing strain‐specific antisera for vaccine potency determination to address a bottleneck that was encountered in preparation of reagents for H7N9 vaccines being developed for clinical evaluation. The combination of traditional bromelain‐cleaved HA and mammalian VLPs for sequential immunization resulted in antisera suitable for use in the standard SRID assay used to determine vaccine potency. The need for alternative approaches and backup techniques for reagent preparation have been recognized previously as an important component of pandemic preparedness. It is also important that the methods used to produce reagents for influenza vaccine testing are documented, particularly in instances when non‐traditional approaches are utilized. The work described here represents the first production of a potency antiserum, which employed such an alternative technique that has been made available by a WHO ERL for general use by vaccine manufacturers and other regulatory agencies.
